# A qualitative exploration of family experiences with a virtual cooking and nutrition programme

**DOI:** 10.1017/S1368980025101717

**Published:** 2025-12-26

**Authors:** Sarah Egan, Amy Saxe-Custack

**Affiliations:** Charles Stewart Mott Department of Public Health, Michigan State University–Hurley Children’s Hospital Pediatric Public Health Initiative, Flint, MI, USA

**Keywords:** Virtual cooking programme, family cooking class, cooking self-efficacy, nutrition education, food delivery

## Abstract

**Objective:**

The current study examined perceptions of and experiences with Flint Families Cook, a virtual cooking and nutrition programme for youth and families.

**Design:**

Families were invited to participate in virtual focus groups after completing the 5-week Flint Families Cook programme. The research study was guided by Social Cognitive Theory. Researchers used thematic analysis to examine the transcribed focus groups, identify patterns across transcripts and develop emerging themes.

**Setting:**

Families living in Flint and surrounding Genesee County, Michigan, USA, engaged in virtual focus groups via Zoom.

**Participants:**

Youth (*n* 32; 59 % female, 53 % African American) and adult caregivers (*n* 31; 90 % female, 39 % African American) participated in focus groups between October 2020 and February 2022.

**Results:**

Five themes were generated from the focus group discussions: (i) general cooking challenges; (ii) class format; (iii) family support; (iv) provision of food; and (v) instruction and learning.

**Conclusions:**

In addition to perceived positive impacts on cooking skills and nutrition education, many participants shared that Flint Families Cook encouraged family cohesion and support. Most caregivers felt the programme, which included instruction by a chef and dietitian as well as ingredient box delivery, had important impacts on the emotional health of youth and family resilience. Flint Families Cook, and similar virtual scalable programmes, could broadly reach children and families to support physical and psychosocial health, especially in low-resource communities where such interventions may be most beneficial.

Most children and adolescents living in the USA consume less than the recommended daily amounts of fruits, vegetables, whole grains and dairy products^(1,2)^. Such deviations from dietary guidelines are associated with poorer health outcomes throughout the lifespan. Diet and diet-related chronic diseases, including heart disease, diabetes and chronic kidney disease, rank among the top causes of disability and death in the USA^(3)^, and substandard dietary intake is the leading cause of death globally^(4)^.

Preparing and consuming home-cooked meals, as opposed to eating meals prepared outside the home, have been associated with healthier diet quality including consumption of less total calories, carbohydrates, fat and sugar^(5)^ and more servings of fruits and vegetables^(6)^. Unfortunately, the USA has experienced a cultural shift away from cooking and eating at home. People devote about half as much time to cooking each day compared to 50 years ago and spend more than half of their food budget on foods eaten away from home^(7)^. Diet quality has likely been impacted by these changes, as foods eaten away from home generally contain more fat, sugar and salt than home-cooked alternatives^(8)^.

Cooking and nutrition programmes have emerged as a strategy to improve diet quality through hands-on culinary instruction paired with nutrition education^(9)^. Beyond teaching culinary skills, these programmes aim to improve food literacy, defined as ‘the knowledge, skills, and behaviours needed to incorporate healthy food into one’s diet to promote lifelong wellbeing’^(10–12)^. Youth culinary and nutrition programmes have gained popularity in part because of the preventive role food literacy education may play in reducing childhood obesity^(11,13)^. Although healthy cooking programmes vary in scope and duration, even short-term exposure has been shown to positively influence youth cooking skills^(14,15)^, cooking attitudes^(16,17)^, diet quality^(13,16–18)^ and health-related quality of life^(16)^.

In recent years, a number of in-person cooking programmes have expanded their reach by launching virtual versions. While some programmes use recorded video lessons to instruct participants^(19,20)^, other virtual classes connect programme staff and participants via an interactive livestream where instructors and participants cook together^(21–26)^. The virtual format requires that all participating households be equipped with the necessary food items and cooking tools to make the class recipes, and participants are generally responsible for acquiring these items on their own^(19,20,22–24)^. Participants must have Internet access and appropriate technology, such as a laptop, tablet or cell phone, to engage with the virtual lessons^(19,20,22–24)^.

Many virtual cooking programmes were launched in the USA during the COVID-19 pandemic when individuals and families were spending most of their time at home^(19–28)^. Preliminary results have suggested that participation in virtual cooking programmes is associated with improvements in cooking attitudes^(21,24)^ and skills^(19–22,26)^. However, little is known about participant experiences with these programmes as well as their perceived impact on individuals and households. This study sought to elucidate perceptions of a virtual cooking and nutrition programme, Flint Families Cook, to understand family experiences.

## Methods

### Study design

This study, guided by Social Cognitive Theory, utilised reflexive thematic analysis^(29)^ to examine perceptions of and experiences with a virtual cooking and nutrition programme for youth and families. The researchers took an interpretivist theoretical perspective in order to capture participant knowledge formed through their social interactions both inside and outside of Flint Families Cook. Researchers assumed a subjective, social constructionist epistemological position that allowed participants and researchers to create knowledge together, participants through sharing their lived experiences and researchers through their interpretation of participant experiences^(30)^. Researchers conducted focus groups with programme participants to collect rich, open-ended data from individuals that engaged in Flint Families Cook together, allowing for researchers to hear individual perspectives as well as shared group experiences.

### Study setting

The current study was implemented in Flint, Michigan, a low-income urban area where approximately 59 % of children live in poverty^(31)^, and a dearth of full-service grocery stores limits access to healthy, fresh, affordable foods^(14,32)^. In March 2020, the surging COVID-19 pandemic presented additional challenges to those living in Flint and surrounding Genesee County. Unemployment rates in Flint swelled from 5·4 % to 29·7 % from March to April 2020^(33)^, and food insecurity in Genesee County rose to 16 % (exceeding the 11·5 % food insecurity rate in the state of Michigan^(34)^). Further complicating food acquisition efforts, residents of Genesee County reported limited selection of foods and inflated prices at grocery stores during the pandemic^(35)^.

### Flint Families Cook

Flint Kids Cook, a healthy in-person cooking and nutrition programme for youth taught in a farmers’ market kitchen, was introduced in Flint in 2017. The programme, which is described in a previous publication^(16)^, was grounded in Social Cognitive Theory and included six consecutive weeks of instruction with recipes and nutrition content highlighting a specific food group. Flint Kids Cook had several active classes underway in March 2020 when the COVID-19 pandemic caused an abrupt halt to all in-person programming. Driven by the growing number of youth on the class waitlist as well as a newfound desire for child-friendly at-home activities^(35)^, researchers developed Flint Families Cook, a virtual cooking and nutrition programme for families^(26)^. Flint Families Cook was modelled after the in-person programme and included 7·5 h of live video instruction from a chef and registered dietitian over five consecutive weeks. From October 2020 through February 2022, a credentialed chef taught healthy recipes from a demonstration kitchen while families followed along from home. Recipe ingredients were delivered to families before class each week by a local food hub (Flint Fresh). A registered dietitian led nutrition activities that highlighted food groups included in recipes. Recipe ingredients, delivery and the 5-week course were free to participants.

### Recruitment and data collection

Families were eligible to participate in Flint Families Cook if there were children in the home between the ages of 8 and 18 years. Family members were also required to speak English, have access to the Internet and a device to join the virtual classroom (such as a laptop, iPad or cell phone) and live in Flint or the surrounding area. Researchers invited all families who completed Flint Families Cook to join virtual focus group discussions which occurred 1 week after the classes had ended (week 6).

Researchers utilised the tenets and constructs of Social Cognitive Theory^(36)^ to develop the focus group guide which explored family experiences with Flint Families Cook as well as dietary behaviours and meal preparation within the home food environment (Table 1). Between November 2020 and February 2022, two researchers trained in qualitative research methods facilitated seventeen focus groups. Three focus groups were co-moderated by both researchers at the beginning of the study, and the remaining groups were moderated by one researcher while a meeting host took notes and audio- and video-recorded the session. After facilitating, moderators debriefed with the researchers who developed this paper. Each focus group included a combination of caregivers and children, with some households represented by only one caregiver or one child and other households having a combination of adults and children together, allowing researchers to explore various perspectives. Both children and caregivers contributed meaningfully to discussions. Children primarily spoke about their experiences during the class, while adults offered insights about class logistics and broader impacts of the class within the home. Focus group discussions lasted approximately 60 min, and families received one $30 gift card for their participation. Researchers concluded data collection when no new responses were offered, and data saturation had been reached. This study received exempt status from the Michigan State University Institutional Review Board. Caregivers provided written consent through a secure digital platform prior to participating in Flint Families Cook, agreeing to take part in the class and share their opinions and experiences upon completion of the programme. Likewise, children provided written assent to participate.

**Table 1.** Relationship between Social Cognitive Theory, concepts and questions

Social Cognitive Theory	Concepts	Research questions	Focus group questions
Environment	Home food environment	To what extent did participation in Flint Families Cook modify the food environment surrounding the family?	What changes, if any, did you notice in the way your family cooks or thinks about cooking since participating in Flint Families Cook?
			What changes, if any, did you notice in the foods your family eats since participating in Flint Families Cook?
	Home mealtime environment		What changes, if any, have you noticed during family mealtime?
	Modelling		When deciding what to feed your family (or eat), how often do you think about healthy food choices?
	Support from others		How, if at all, did your family support one another while participating in Flint Families Cook?
Person	Knowledge	How do caregivers and youth perceive their own ability to prepare and consume healthy foods following participation in Flint Families Cook?	What, if anything, did you learn about nutrition while participating in Flint Families Cook?
			What, if anything, did you learn about cooking while participating in Flint Families Cook?
	Skills		Please describe any cooking skills that you learned in the class that have been practised at home.
			How, if at all, did the class change the way you feel about cooking?
	Outcome expectancies		Do you think you will continue to cook at home together now that Flint Families Cook has ended? Please explain.

### Data analysis

Reflexive thematic analysis was used to analyse the focus group data. Focus group discussions were transcribed verbatim. Two researchers followed a multi-phase inductive analysis process to develop meaning from the dataset^(29)^. After familiarising themselves with the data, the researchers independently coded the data and used code labels to generate initial themes that were representative of core ideas in the dataset. Together they consolidated their initially generated themes to develop themes and sub-themes that highlighted key patterns found in the dataset that were relevant to the research question. They returned to the transcripts to refine the themes and sub-themes until they felt true to the dataset. Lastly, researchers selected illustrative quotes to support each theme and sub-theme and wrote up the analysis.

### Reflexivity

The authors were mindful to practise reflexivity through ongoing collaboration, returning to the dataset throughout the process to check that the analysis faithfully represented the transcripts and addressed the research questions. Authors immersed themselves in the data to interpret patterns beyond the explicit responses to the focus group questions.

## Results

Between October 2020 and February 2022, 153 youth (mean age 11·28 ± 2·36 years) from 104 families participated in Flint Families Cook. The majority of youth identified as female (58 %), African American (69 %) and residents of Flint (75 %). A total of sixty-three participants (thirty-one adults and thirty-two youth) engaged in seventeen virtual focus groups. Similar to programme participants, the majority of youth who participated in focus group discussions identified as female (59 %), African American (53 %) and residents of Flint (81 %). Among adult focus group participants, most identified as female (90 %) and lived in Flint (71 %). Approximately 39 % identified as African American and 39 % identified as Caucasian. Five themes were generated from the focus group discussions: (i) general cooking challenges; (ii) class format; (iii) family support; (iv) provision of food; and (v) instruction and learning (Table 2).

**Table 2.** Illustrative quotes collected from Flint Families Cook participants

Theme	Sub-theme	Quote
1. General Cooking Challenges	1·1 Clean-up	‘The space and then the mess after and stuff like that, trying to get it all put back together’. (Caucasian female caregiver, age 33)
		‘Cleaning up. [My child] does not like that part’. (African American female caregiver, age 40)
		‘I want to just go sit down and have somebody wait on me, so I don’t have to do the dishes’. (Caucasian female caregiver, age 43)
	1·2 Time	‘Sometimes I don’t want to make [recipes] because it takes so long to bake’. (Caucasian female caregiver, age 43)
		‘Time, time constraints. We have very, very busy schedules’. (Other racial female caregiver, age 37)
		‘Making time so that they can all participate. We are so busy sometimes’. (Other racial female caregiver, age 40)
	1·3 Cooking tools	‘Some people don’t have all the cooking supplies that you might need like spoons, tin foil, and just stuff that you might need on the fly’. (Caucasian female caregiver, age 43)
		‘Sometimes if we have to use measuring cups, not being able to find certain ones [is a challenge]’. (Caucasian female youth, age 13)
	1·4 Food preferences	‘Making a recipe that everybody’s going to eat because sometimes you have so many different opinions on certain things and people are picky about stuff’. (Caucasian male caregiver, age 52)
		‘Sometimes I pick stuff [my kids] don’t like, and then they don’t want to eat it and then I’m like, “Well I wasted all this time cooking and now nobody wants to eat it”’. (Caucasian female caregiver, age 43)
2. Class Format	2·1 Time and Length	‘One night a week, that was a huge relief for me. Okay [my son is] making dinner, I didn’t have to shop. I didn’t have to add things to the list, that was nice’. (Caucasian female caregiver, age 34)
		‘I feel as if [the class was at] a perfect time because there’s a lot of kids with parents who work and make it home at 5 o’clock. And they’re there to do family bonding like me and my kids’. (African American female caregiver, age 36)
		‘The time of the day, and the regularity of it all, starts putting you in a structure… you start getting into a good habit of cooking meals around this time’. (Hispanic male caregiver, age 48)
	2·2 Multiple Class Nights	‘I liked that I could switch to either day… Sometimes you think, ‘Oh I’m going to be home every Wednesday’ and then stuff comes up. I like that you have the flexibility of going to a different day’. (Caucasian female caregiver, age 43)
		‘It was nice because if we couldn’t do certain things on one night, we were always able to do it on the other’. (Caucasian female youth, age 13)
		‘[If] we did miss a Wednesday, we could do it on a Thursday because we did have something that came up. It was really easy that if we miss something we could always do it another day, and that made it really convenient for us’. (Other racial female caregiver, age 40)
	2·3 Engagement	‘I think this is a great resource for a lot of people and kids in this time’. (Caucasian female caregiver, age 34)
		‘My daughter is very conscious of COVID. She’s just always thinking about COVID. She wears her mask all the time, so to be able to participate in something that didn’t require her to think really hard like schoolwork and it was fun and exciting and engaging with her family, she really appreciated that’. (Other racial female caregiver, age 50)
	2·4 Technology	‘I had some internet difficulties… we had some storm days and then we ended up missing a session because the internet went out in the afternoon’. (African American female caregiver, age 41)
		‘My computer is small, so we just got a bird eye view. And I’ll be over at the stove trying to look over my shoulder at the computer, and I just couldn’t see that well. So, it just didn’t flow that well because of the visual’. (African American female caregiver, age 37)
		‘It was hard for me to hear sometimes, I had to hook up to a speaker at home. So then just background noise at home was hard for me too because my fan was going on my oven, and my daughter was talking and then I’m like, “Oh shoot, I don’t know what step they did next”’. (Caucasian female caregiver, age 43)
	2·5 Class Pace	‘[My son] struggled a little bit with the timing. I think he’d just get flustered if he wasn’t going as fast and then he’d start messing up’. (Caucasian female caregiver, age 33)
		‘Maybe a possible suggestion for the future, there could be different class sessions for if you’re a beginner, if you’re a moderate…and they can go at different paces. That way people don’t have to be either going too fast or going too slow, it can just be just right for you’. (Multi-racial male youth, age 17)
		‘Sometimes I would be rushing because I wouldn’t be able to do this a certain way, or I would have to go get a certain measuring cup or some type of spoon or whisk… I felt like [the chef] was doing the directions too fast’. (Caucasian female youth, age 13)
	2·6 Socialisation	‘I think that would have made it better for [my son] if other kids got to converse amongst each other, but some kids don’t want to or aren’t comfortable with it’. (Caucasian female caregiver, age 34)
		‘It would be so much easier if it were in-person. I know the pandemic and everything now, but us being at home, I don’t think we got much of a connection at all with other families, except for we got to see familiar faces every week. That’s about it’. (Caucasian female caregiver, age 36)
		‘I think that [an in-person class] would have a good value especially for the kids that really do enjoy cooking. They might find another kid that they can relate to and be friends with outside that they can have that similar interest with as well’. (Caucasian female caregiver, age 33)
3. Family Support	3·1 Class Preparation	‘I thought the prep steps were a good idea because it made things a lot easier. And I think [my daughter] was able to enjoy it more being prepped ahead of time’. (African American female caregiver, age 47)
		‘Everything was itemized, so we were able to see what we had and didn’t have and if there were some things that we didn’t have, I tried to make sure, before we got on, okay do we have everything? And if we don’t, what can we substitute as far as tools?’ (African American female caregiver, age 50)
		‘We would get [the prep steps] done like a day or two ahead. Whenever we got the box, that’s when we did the prep steps’. (African American female caregiver, age 39)
	3·2 Teamwork	‘My girls can hardly ever get along, but they were working together during the class. They were being nice to each other. I was like, “Oh my goodness we should do this every week!” Because they were actually using teamwork. That was kind of unusual but great’. (Caucasian female caregiver, age 36)
		‘Her dad would come home, right after all the food was made when everything was coming out of the oven. He’d be the first one to sample everything. Then she has an older sister and a little sister who also like sampling the food so the whole family kind of worked together’. (Caucasian female caregiver, age 42)
		‘It was me and my father who were doing [the class] and my stepmother and even my stepsisters help if we had work or school and we’re coming late, they would get some of the materials out for us, and they helped taste test for us too. So it was like the whole household’. (Multi-racial male youth, age 17)
	3·3 Family Bonding	‘I think [my daughter] was very excited, just about the family bonding. The fact that, I could see her smiling, like “Come on it’s time for us to cook,” and the box would come, I could just see the happiness in her face in the fact that we were doing this together’. (Other racial female caregiver, age 50)
		‘[My daughter] and I would do the first [recipe], and my son and [wife] would do the second one so we would swap it out. [My daughter] and I bonded over this, this has been a lot of fun, we had a great time’. (Caucasian male caregiver, age 52)
		‘We have my stepson week on and then his mom has him week off. So, for the five weeks, he was at our house one week then her house another week. That was nice, one day she actually came over and cooked at our house with him, so we kind of came together there’. (Caucasian female caregiver, age 33)
4. Provision of Food	4·1 Delivery	‘[The ingredient box] came on a Tuesday, and [my kids] were able to look over it and see all the ingredients and everything, so they were able to cook it Thursday. It came in time to make sure they had everything’. (African American female caregiver, age 36)
		‘It was nice because they just ring the doorbell and gave us the box, and it was neatly packed and everything. And it was fancy, my mom said’. (Caucasian male youth, age 15)
		‘I really like how you delivered too. That was really cool. We’ve never had groceries delivered so the kids are excited… everything was good food, nothing was bad about it. You always wonder when people pick your groceries for you what they pick out, but they always sent us really good stuff’. (Other racial female caregiver, age 40)
	4·2 Quality and Packaging	‘I just love how they included every single ingredient, even the laminated recipe card. I love how they included, even down to the salt and pepper’. (African American female caregiver, age 37)
		‘The quality was really great. I was surprised on how much was there, it was a lot! It fed all four of us, and sometimes had leftovers’. (Other racial female caregiver, age 40)
		‘Everything was well packed and organized with the seasonings… I thought they did a very, very good job with how they package things arranged and measured amount and everything’. (African American female caregiver, age 41)
	4·3 Child Excitement	‘The kids were always excited when the box came. When they got home from school, they were excited to look in there. See what was in there, check it all out, and look at the recipes, see what they were making that week’. (Caucasian female caregiver, age 30)
		‘[The ingredient box] kind of aroused [my kids’] curiosity too because they’re like, “What is this?” It made them want to be involved or know more about it’. (Hispanic male caregiver, age 48)
		‘[My kids] were excited when we knew the box was coming to see what was in it because I didn’t tell them what we were making the next week. So, when we got the box, we figured out what we were making, and they would look over the recipe and see what was in the box’. (Other racial female caregiver, age 40)
5. Instruction and Learning	5·1 Cooking Skills	‘Even my husband and I learned new things. I’m like, “Okay I guess, I don’t even know how to chop a carrot.” We weren’t dicing an onion correctly, so we learned new tidbits of stuff’. (Caucasian female caregiver, age 43)
		‘I used to be afraid of the cutting board and the knife because I was afraid I was going to cut myself with it, but now that I’m done [with the class], I’m more comfortable around the knife and using the cutting board with an adult, or someone older around to help me’. (African American female youth, age 8)
		‘I feel more comfortable with [my son] being in the kitchen, and he’ll initiate things now, more so than before. He’s always wanted to help, but I think I’ve been more reluctant to let an eight-year-old help thinking that he wasn’t quite ready. And this has proven to me that he is responsible enough. I mean he still needs help and assistance, but I feel comfortable with giving him tasks beyond being a runner’. (African American female caregiver, age 50)
	5·2 Nutrition Education	‘I thought [the nutrition education] was helpful. It reiterated some things that we’ve heard from nutritionists before and definitely put it into practical terms for a preteen’. (African American female caregiver, age 41)
		‘I thought [the nutrition lesson] was very educational and stuff that I needed to know personally because it’s going to help me with my health in my future’. (African American female youth, age 12)
		‘You don’t have to get your protein from meat and stuff. You can get it from other [sources] like beans’. (Caucasian female youth, age 17)
	5·3 Food Exploration	‘[My daughter is] really into fresh now so we go to the farmers’ market, and she picks up her fresh fruits and veggies and stuff that she wants to try, and I can see a huge difference in just her every day, even more willing to try different things now’. (African American female caregiver, age 47)
		‘I didn’t really like vegetables and healthy-like foods… at first when I heard about fruit rice or even whole wheat tortillas, I was traumatized, I was like, “What are we going to be eating? I’m not going to eat that.” But after trying it, I loved it, and now that I like it, I’m definitely going to make it again, and it really built confidence just to try out other foods that you didn’t think you’d like before’. (Multi-racial male youth, age 17)
		‘I would have never, not in a million years, thought to squeeze orange juice on green beans when we did the teriyaki green beans that day. And they were actually delicious. [My daughter] cooked it again the next day. I went and got her some more ingredients and she made it again the next day because we enjoyed it’. (African American female caregiver, age 47)
	5·4 Outlook on Cooking	‘Look how many things that we made just in that short time span during the class. So, it’s my wish and my focus that the kids and I [cook at home] more often’. (Caucasian female caregiver, age 43)
		‘We haven’t cooked together in quite some time, so it was really nice, and I also think that this isn’t something that just happened during that 6-week period, I think it’s something that we’re going to continue doing as we go forward’. (Other racial female caregiver, age 50)
		‘Yeah, [cooking at home], it’s not that hard. Not as hard as I thought it was’. (Caucasian female caregiver, age 42)

### Theme 1. General cooking challenges

Many caregivers discussed general barriers that prevented regular meal preparation at home. Even among a population interested in cooking, focus group families spoke about important factors that hindered their ability or desire to cook at home. Some of these challenges included lack of time or motivation to cook, while others were related to a limited supply of cooking utensils in the home.

#### Sub-theme 1.1. Clean-up

Some focus group participants specifically noted that the *clean-up* required after preparing a meal contributed to a lack of cooking at home. Simply the thought of cleaning the kitchen was frequently described as a deterrent to cooking.

*I love the cooking. I don’t like the clean-up. (Caucasian female caregiver, age 50)*

#### Sub-theme 1.2. Time

Many families expressed a desire to regularly cook meals at home but were challenged with adequate *time* to prepare meals with their families. Lack of time was often related to busy household schedules, including both child and adult activities outside the home, that limited time spent in the kitchen. Some simply perceived the act of cooking in the home to take considerable time.

*Time [is a challenge], and just generally a lot of things going on. Some days we might not cook something as elaborate, we might make something that you just have to heat up. (Caucasian female caregiver, age 37)*

#### Sub-theme 1.3. Cooking tools

Most families new to cooking noted that without the necessary *cooking tools*, it was difficult to prepare recipes. Some shared that when kitchen supplies were unavailable, they felt limited in the types of foods they could prepare. Many caregivers and children mentioned the particular importance of having measuring cups and spoons available when following recipes.

*If you have the bare minimum when it comes to kitchen tools and supplies, that could be challenging when it comes to getting the precise measurements. (African American female caregiver, age 41)*

#### Sub-theme 1.4. Food preferences

When cooking for multiple household members, some focus group participants expressed frustration with accommodating various *food preferences*. Many caregivers voiced difficulty finding recipes that all members of the household would agree upon and enjoy. Others seemed discouraged when describing meals they had prepared at home that all household members would not eat.

*Sometimes we all like different things, so that’s where it can be hard. Some people don’t like potatoes or my husband [doesn’t like] peas. It’s just hard to find something that everybody likes. (Caucasian female caregiver, age 43)*

### Theme 2. Class format

Flint Families Cook included 7·5 h of live video instruction from a chef and registered dietitian over 5 weeks. Classes started in the evening at approximately 5:00 PM. While the virtual format of the class offered benefits, the reliance on technology also introduced obstacles.

#### Sub-theme 2.1. Time and length

Most families expressed appreciation for both the *timing* of classes, which occurred after regular work hours and during dinnertime, and the *length* of classes, which seemed appropriate to engage youth and families.

*Me and [my daughter] really liked the Zoom classes because as soon as I got home from work, we could just go straight into the cooking class. And she had a few minutes after school to prep whatever was needed. (Caucasian female caregiver, age 42)*

#### Sub-theme 2.2. Multiple class nights

Classes were offered two nights each week, and families were given the option of attending the class that best fit their schedules. Most families appreciated the flexibility in class schedules and noted that *multiple class nights* prevented unpredictable factors, such as technology malfunctions or schedule conflicts, from causing class absences.

*Having the option for two days was really good, because there were days where I could do one day or the other and same thing with my family. For the older participants especially, if you have extracurriculars or anything, I had work some days, it was really good having that flexibility. (Multi-racial male youth, age 17)*

#### Sub-theme 2.3. Engagement

During the COVID-19 pandemic, many children, unable to participate in usual after-school activities, were looking for new ways to spend their time. Most families expressed gratitude for Flint Families Cook, an *engaging activity* for families during this time that served as a resource for youth and families.

*With COVID going on, it gave the kids something to do…It’s pretty awesome for them to have a class that they can focus on besides school, and that they can enjoy. Something that was able to de-stress them, knowing that there’s so much craziness going on with COVID. (African American female caregiver, age 36)*

#### Sub-theme 2.4. Technology

On the other hand, the virtual programme format also presented challenges for some families. Because classes relied on *technolo*gy, attendance and participation were directly impacted by power outages and disrupted Internet connections. Additionally, many families discussed frustrations with the individual devices used to access the classes, including struggles with viewing the computer screen or hearing instructions during recipe preparation.

*Some of the weeks I accessed [the class] on my phone. Some weeks I accessed it on my tablet. The internet service has been kind of spotty back and forth. (African American female caregiver, age 57)*

#### Sub-theme 2.5. Class pace

Many caregivers further mentioned that the *class pace* was not suitable for all families and should be addressed. Some shared that the chef moved through the recipes too quickly and requested that the class pace be slowed. Others suggested stratifying classes in accordance with household cooking experience to help keep all participants moving through the class at the same pace.

*There was a couple of times when [the chef] got a little bit ahead of us. So, we were trying to catch up. (Caucasian male caregiver, age 52)*

#### Sub-theme 2.6. Socialisation

Caregivers and children shared that the class format limited *socialisation* between families. Many caregivers noted that youth, with a special interest in cooking, might have preferred cooking with other children in the class. Some participants also noted that it was difficult to connect with other families during the virtual classes when many had their microphones muted or screens hidden.

*I don’t think we really connected with the other families… just because you were at home with your family. And I think if we were to come in and take a cooking class, my child would be more apt to be friendly with the kids. I think just because it’s online, it’s a little bit harder.* (*Other racial female caregiver, age 40*)

### Theme 3. Family support

Successfully implementing Flint Families Cook within 104 unique households required consistent investment from caregivers as well as other family members in the home. Focus group participants shared how they supported their family members both before and during the class.

#### Sub-theme 3.1. Class preparation

Many caregivers discussed how their families engaged in *class preparation* each week to ensure that they were ready with cooking tools, food and technology. Some shared how they carefully reviewed recipes to determine which cooking utensils and ingredients were required. Others talked specifically about food preparation steps, referred to as ‘prep steps’ within the curriculum. These ‘prep steps’ involved washing, peeling and cutting produce in preparation for classes. Families were encouraged to complete these steps before class to avoid feeling rushed once live instruction started.

*I would try to prep beforehand with [my daughter] like, ‘Let’s find all the measuring cups we’re going to need, all the ingredients we’re going to need, what spoons, what bowls’. (Caucasian female caregiver, age 37)*

#### Sub-theme 3.2. Teamwork

Focus group participants shared how family members came together to practise *teamwork* during the classes. For some, this meant delegating age-appropriate tasks to siblings during recipe preparation, while others shared a genuine feeling of cooperation and support between siblings during class. Members of the household who did not actively participate in cooking often shared in the experience through tasting the prepared meals and offering feedback and support.

*The older two [kids] got to do the cutting which I know they liked because they learned new techniques… they took turns a lot and if the older two got to cut, the little one got to mix or measure or things like that. (Other racial female caregiver, age 40)*

#### Sub-theme 3.3. Family bonding

Most families talked about the value of Flint Families Cook in creating a special time for *family bonding*. Some shared discussions they had with their children while preparing recipes, while others appreciated that this was a dedicated time, free from distractions, for families to simply be together. Some caregivers further mentioned that extended family and friends, who may not live in the home, joined the classes with their children. Various situations were described by participants, including families with shared custody of children who came together to cook; grandparents and other family members who helped caregivers when they were unable to make a class; and youth who invited friends over to cook with them.

*It gave us some very dedicated quality time together. It was a time that we were together, intentionally. At home, not work, not school, not video games, not TVs, we’re right here focused on the same task, really working together and creating some memories. (African American female caregiver, age 41)*

### Theme 4. Provision of food

Flint Families Cook was introduced while grocery store shelves remained empty and food supplies were scarce. Flint Fresh, a food aggregation space for local farmers with mobile food delivery services, partnered with the programme to provide families with ingredients to prepare the class recipes each week. During focus group discussions, it was clear that this part of the programme was critical to its success.

#### Sub-theme 4.1. Delivery

Families spoke highly of Flint Fresh, with noted comments about positive experiences with the *delivery* service. Focus group participants appreciated the convenience of receiving food boxes at their homes and had pleasant experiences with the delivery drivers.

*The delivery people were really good. They would call me at work and say, ‘Hey, we’re getting ready to come to your house. Is it okay, is there anybody home?’ So, that was nice that I didn’t have food sitting out on my porch getting yucky because of the heat. (Caucasian female caregiver, age 43)*

#### Sub-theme 4.2. Quality and packaging

Participating families noted that the *quality and packaging* of the food in the ingredient box exceeded their expectations. Families were impressed with the dry ingredients in vacuum-sealed packages, fresh produce and generous portions of food provided in their ingredient boxes.

*The boxes were good, a great setup. We were here to bring it in right away and put it away. And there was enough to make the meal and maybe have a little bit extra. I thought all of the packaging was well done, and all the food was fresh and healthy and didn’t have anything that was over-ripe or bad or anything like that. (Caucasian female caregiver, age 42)*

#### Sub-theme 4.3. Child excitement

Many caregivers shared that simply receiving the ingredient box each week elicited *excitement from children*. Some children talked about anxiously awaiting the arrival of the ingredient boxes to see what was inside, while many caregivers shared that the boxes motivated children to want to cook. Several caregivers noted that the ingredient boxes were the highlight of the programme for their children.

*It was exciting because it felt like a Christmas gift that you were waiting to open every single week…we were so excited to see what we were going to make. (African American female youth, age 12)*

### Theme 5. Instruction and learning

Most focus group participants discussed how the classes offered practical culinary and nutrition education as well as activities that encouraged food exploration. Through the preparation of the weekly recipes with the chef and nutrition education lessons led by the registered dietitian, most families felt they had learned how to identify and create healthy meals.

#### Sub-theme 5.1. Cooking skills

Children and adults shared that the programme taught or improved their *cooking skills*. Many participants learned proper knife skills and how to measure recipe ingredients. Several children expressed improved confidence in their cooking abilities, while caregivers shared how they feel more comfortable with their children cooking at home because of their growth during the programme.

*[My daughter] definitely learned how to cut, because that was something I didn’t let her do before. But I’ve seen that she was good at it, and she took her time. So, that was great, and she’s learned how to measure ingredients and to look at the label on the measuring spoons and measuring cups. (Caucasian female caregiver, age 30)*

#### Sub-theme 5.2. Nutrition education

Each class included a nutrition lesson facilitated by a registered dietitian. Nutrition content was directly related to the food group that was featured in recipes each week. Families appreciated the discussions and interactive activities that provided *nutrition education* for the entire family.

*You learn from the chef, but you also learn from [the dietitian]. She broke it down into the different food groups and taught about each food group and why it’s good for your body. I kind of knew some of that stuff too but even I learned things. (Caucasian female caregiver, age 43)*

#### Sub-theme 5.3. Food exploration

The recipes prepared in class featured a variety of healthy ingredients that encouraged *exploration* among participants. In their ingredient boxes, families were introduced to new fruits, vegetables, whole grains and low-fat dairy products. Some participants tried new ingredients for the first time, while others explored recipe preparation with new foods. Each of the class recipes included healthy substitutions, such as incorporating more servings of vegetables, swapping whole grains in place of refined grains or naturally sweetening recipes with fruit or honey.

*[The kids] did get introduced [to] new foods, as well as different alternatives of how we traditionally make them, like the mac and cheese was totally not how we make it, but it was good. Everything, all the recipes were amazing. They put a different light on the actual ingredients and how they turned out, so it’s good to have some alternatives, and they still taste great. (African American female caregiver, age 49)*

#### Sub-theme 5.4. Outlook on cooking

Prior to taking the class, many families aspired to regularly cook meals at home, but most felt that their goals were unrealistic given their lifestyle and skillset. After completing Flint Families Cook, families expressed an improved *outlook on cooking*. For some, this meant cooking more regularly or intentionally including children in meal preparation. Others spoke about how the class improved their perception of cooking.

*I got all kinds of confidence…Before I used to be intimidated by the word ‘cook’ and if anyone ever said, ‘Can you help me prepare this food?’ I’d get nervous about using the stove and measuring and how things would turn out, but ever since we’ve done this class, now I feel like I got guts to do anything. Like to make a whole filet mignon dinner. (Multi-racial male youth, age 17)*

## Discussion

Flint Families Cook was successful in delivering cooking and nutrition education to families virtually, but perhaps more notable was the sentiment that the programme facilitated family bonding. Consistent with earlier findings related to an in-person parent–child cooking programme^(37)^, both caregivers and children in the current study enjoyed spending quality time with their families during class. Families spoke about how Flint Families Cook encouraged them to support one another and work as a team. Moreover, some talked about the inclusion of extended family members and friends in the programme. Research has shown that involving family members in shared activities, such as preparing and eating meals together, helps to increase family unity and resilience, particularly during times of significant stress^(38,39)^. The COVID-19 pandemic deeply affected households with children, as many schools, daycare centres and work locations were impacted by stay-at-home orders^(28)^. Social isolation, fear of illness, economic hardship and reduced psychological supports contributed to worsened mental health for both adults^(28)^ and youth^(40)^. Studies conducted during the COVID-19 pandemic reported that youth were less likely to exhibit depressive symptoms when their family maintained consistent routines and shared positive experiences together^(40,41)^. When families gathered in their kitchens each week to cook and share a meal, they were likely protecting one another from adversity inflicted by the pandemic^(38)^. Furthermore, while most youth recorded declining quality of life during the pandemic^(42)^, previous research has suggested that young Flint Families Cook participants experienced improvements in health-related quality of life during the 5-week class^(43)^. This is in concurrence with results found in another virtual cooking programme offered to children during the COVID-19 pandemic that reported improvements in child wellbeing upon completion of the programme^(44)^. Although Flint Families Cook was designed to improve cooking and nutrition, the programme offered recurring opportunities for families to connect and work together during a difficult time.

The provision of ingredient boxes, delivered each week by Flint Fresh, was a key feature of Flint Families Cook. While some virtual programmes offer vouchers to subsidise class ingredients^(20,23)^, Flint Families Cook was unique in supplying complimentary home-delivered food boxes that included all necessary ingredients. Families praised the convenience of the delivered boxes, especially because of the elevated costs and limited availability of many grocery items during the pandemic^(35)^. Inability to access or afford foods is a noted deterrent to participating in virtual cooking classes^(23)^. In addition to increasing programme accessibility, the delivered boxes introduced families to healthy ingredients they otherwise may not have purchased due to lack of familiarity or financial constraints^(45,46)^. Both adults and youth discussed how they incorporated many new foods created from these ingredients into their diets, an outcome reported in other virtual cooking programmes^(24,47,48)^.

While participating in Flint Families Cook, each caregiver was responsible for ensuring the successful implementation of the virtual programme within their home. Caregivers made decisions that were appropriate for the unique needs and preferences of their household. Most caregivers spoke about how they prepared their kitchen before each class by assembling cooking equipment, following the food ‘prep steps’, and setting up technology. This pre-class set-up contributed to a positive class experience. Previous research focused on virtual classes has noted that adequate preparation before class was paramount to success^(25)^. Many caregivers described how they assigned cooking tasks to family members in accordance with cooking skills and facilitated teamwork to ensure that everyone was involved. In some households, caregivers shared that they stepped back and watched as children led food preparation activities. Overall, caregivers seemed to view their role as guiding children during classes, such as making adjustments to speed up the cooking progress when falling behind the pace of the class or troubleshooting when conflicts arose. In these ways, the virtual format required notable commitment from caregivers. This commitment is unlike in-person family cooking classes that typically involve programme staff who prepare the kitchen space, enforce safety and model techniques to include children in the kitchen, allowing caregivers to focus on cooking with their children^(49,50)^. It is unclear; however, whether families who complete in-person cooking classes are successfully able to recreate the cooking environment they experienced during the class in their own homes^(49)^. For example, after finishing a 6-week family cooking class, most caregivers shared that they had not cooked with their children at home because they were concerned about the additional time and vigilance required to keep their children safe in the kitchen^(51)^. Alternatively, Flint Families Cook families practised cooking together at home each week during class, while programme staff provided assistance and encouragement through Zoom. In this way, Flint Families Cook offered structured and supported household cooking time, which may help families continue cooking together at home after the class.

Focus group participants suggested that Flint Families Cook helped overcome barriers to cooking at home. When asked about cooking habits before the class, many caregivers listed various challenges that prevented them from preparing home-cooked meals. Interestingly, after the 5-week class, families’ perceptions of cooking seemed to shift. Most focus group participants believed that cooking at home, especially with their family, seemed more attainable after engaging in the programme. Previous research focused on in-person family cooking classes supports the current findings. After caregivers cooked with their children, caregivers who were hesitant to include their children in the kitchen learned to recognise how children can help in meaningful ways^(37,49,50)^. Some caregivers remarked that cooking at home seemed easier than their previous conceptions and that they aspired to continue cooking together as a family.

Focus group participants pointed to several ways in which Flint Families Cook helped reframe their perceptions of cooking at home. First, the class provided dedicated cooking time each week, which showed busy families how preparing dinner could fit into their schedule. Additionally, families noted improvements in their cooking skills after taking the class, consistent with findings from other virtual programmes^(19–21,26)^, which may increase their efficiency in the kitchen. Adults spoke about learning time-saving tricks from the chef and witnessing their children gain confidence and aptitude in the kitchen. Caregivers often worry that involving children in the kitchen will increase the cooking time^(51)^, but studies have shown that caregivers are more willing to welcome children into the kitchen when they witness their children learning new cooking skills^(49,52)^. Lastly, Flint Families Cook helped families that were challenged to create meals that addressed various household food preferences by introducing them to new recipes. Many focus group participants offered positive feedback regarding class recipes, and some shared how they had incorporated recipes into their family meals. Likewise, participants in other virtual and in-person programmes have reported preparing class recipes again at home^(21,23,37)^. By holding time and supporting families as they cooked together, most focus group participants felt that Flint Families Cook helped address challenges with cooking at home.

This study contains several strengths and limitations. A strength of this study was the inclusion of caregivers and youth in the focus groups, which allowed researchers to hear various participant perspectives. Further, the design of the current study allowed researchers to capture family experiences during a time of crisis and isolation using online methods. One limitation was the small sample of individuals from one low-income community. Because of this, the results may not be generalisable to other communities. Additionally, there may have been selection bias as families that chose to participate in focus group discussions and provide feedback may have had different experiences than those who chose not to participate. However, participants were candid about programme successes as well as programme challenges and offered thoughtful suggestions for improvements.

## Conclusion

In addition to perceived positive impacts on cooking skills and nutrition education, many participants shared that Flint Families Cook encouraged family cohesion and support. Most caregivers felt the programme, which included instruction by a chef and dietitian as well as ingredient box delivery, had important impacts on the emotional health of youth and family resilience. Flint Families Cook, and similar virtual scalable programmes, could broadly reach children and families to support physical and psychosocial health, especially in low-resource communities where such interventions may be most beneficial.
